# Projecting supply and demand for pharmacists in pharmacies based on the number of prescriptions and system dynamics modeling

**DOI:** 10.1186/s12960-020-00524-5

**Published:** 2020-11-05

**Authors:** Yasuhiro Morii, Seiichi Furuta, Tomoki Ishikawa, Kensuke Fujiwara, Hiroko Yamashina, Katsuhiko Ogasawara

**Affiliations:** 1 Faculty of Health Sciences, Hokkaido University, Sapporo, Japan; 2 The Department of Pharmacy, Hokkaido University of Science, Sapporo, Japan; 3 Institute for Health Economics and Policy, Tokyo, Japan; 4 Graduate School of Commerce, Otaru University of Commerce, Otaru, Japan

**Keywords:** Health human resourcing, Pharmacists, Forecasting, System dynamics modeling

## Abstract

**Background:**

Pharmacists play an important role in promoting people’s health in Japan, which has an aging population. Hence, it is necessary that the distribution of pharmacists meets the population’s needs in each region. This study projects the future supply and demand for pharmacists in pharmacies to consider an optimal distribution of pharmacists.

**Methods:**

The future supply of pharmacists working in pharmacies in Hokkaido is projected using system dynamics modeling, according to their career path. The demand is projected based on the number of prescriptions, sourced from publicly available sources. The analysis period is 2015–2040. The estimated demand is converted into the number of pharmacists and the sufficiency is evaluated using sufficiency ratio (supply/demand ratio). Sensitivity analyses of the sufficiency ratio were conducted to estimate the effects of changes in parameters such as national exam pass rate, enrollments, attrition rates, the number of prescriptions per pharmacist, and diffusion of newly licensed pharmacists.

**Results:**

The projected supply, in 2025 and 2040, is 1.24 and 1.56 times, respectively, as that in 2015 and the demand is 1.11 and 0.98 times, respectively. In 2015, although the sufficiency ratio in Hokkaido overall is 1.19, the ratios are higher in urban medical areas and lower than 1 in rural medical areas, such as Minamihiyama, Emmon, and Nemuro. By 2040, the sufficiency ratios are greater than 1 for all areas except for Emmon and higher than 2 in some areas. The sensitivity analyses found that the sufficiency ratio was most sensitive to diffusion of newly licensed pharmacists and the number of prescriptions per pharmacist.

**Conclusion:**

Optimal distribution should be considered, as the results reveal a possible shortage in the number of pharmacists in rural medical areas in 2015–2025. Conversely, as the demand is projected to decrease after 2025 with a population decrease, future supply should be determined in order not to cause an oversupply after 2025. Refinements of the projection model should be conducted since the related factors such as the roles of pharmacists will change over time.

## Background

Japan has the most aged population in the world. Pharmacists in pharmacies in Japan have broadened their roles to include drug/medicine administration instructions, prescription-related questions to doctors, and promoting self-medication, and home medical care [1]. To address the aging society and population decrease, Japan has been establishing a policy called “community-based integrated care systems” where people can have access comprehensively to healthcare services, nursing care, disease prevention, housing, and livelihood support [2]. The system has been established by municipalities and prefectures, which administrate the healthcare insurances, by 2025, when baby boomers will be over 75 years of age. In this system, pharmacies must work as family pharmacies to promote local people’s health [3] as well as simply prescribing. Hence, it is necessary to have a clear vision regarding the future supply and demand for pharmacists to meet the needs in each area. Sano et al. mentioned the possibility of a shortage in the number of pharmacists in certain prefectures and in Japan overall [4]. Morii et al. reported that pharmacists and doctors are unevenly distributed in Hokkaido [5]. Conversely, Hasegawa et al. mentioned a possibility of pharmacist oversupply during the 2020s [6]. There is no consensus regarding whether the current supply of pharmacists is sufficient. Moreover, although there have been reports on the supply and demand for pharmacists, few studies have focused on projecting their supply and demand in Japan. In Hokkaido, which has the largest land area and an aged population [7, 8], disparities in medical resources between regions have been reported [9, 10]; hence, considering an optimal supply based on expected demand in Hokkaido is especially important.

System dynamics (SD) was developed by Forrester in the 1950s [11] to project the quantum of required resources. SD has been applied to diverse fields, such as the environment and administration and to predict the required medical resources [12–14]. For example, Barber et al. projected the supply and demand for specialists in Spain [15] and Ansah et al. projected the supply and demand for ophthalmologists in Singapore [16]. In Japan, SD modeling has been used to project the supply of physicians [17], radiologic technologists [18], and physical therapists [19]. SD is a modeling method based on actual causal relationship (i.e. pharmacists’ career path). The merit of using SD is that SD modeling is not dependent on past data like other statistical methods such as regression analysis, and therefore can reflect recent trend of related parameters. The merit can help policymakers understand what will be expected with the current trend of related parameters (e.g. enrollments, the number of prescriptions per pharmacist per day), and what is expected by potential measures they take (e.g. increase in national exam rate or enrollments and decrease in attrition rates). Another merit is that complex relationships can be simulated using SD. Although supply of pharmacists changes non-linearly because of multi-way paths caused by reexam, entering graduate schools, attrition and so on, these relationships of the related parameters can be simulated using SD.

Prescribing (making prescriptions, dispensing medication, and taking medication history) accounted for approximately 80%, a major part of pharmacists’ work [20]; hence, the number of prescriptions can be considered a proxy of pharmacists’ workload (one pharmacist can deal with a maximum of 40 prescriptions per day under the current legislation [21]). Therefore, the supply and demand for pharmacists in pharmacies can be projected based on the number of prescriptions generated. However, there is little research that evaluates the current supply of pharmacists and projects their future supply using number of prescriptions in Japan. There is also little research related to supply of and demand for pharmacists using SD. This study projects the future supply of pharmacists working in pharmacies using SD modeling simulation, based on the number of prescriptions, to provide data to help determine their optimal distribution.

## Methods

### Subjects and outcomes

The subjects are pharmacists working in the pharmacies of Hokkaido, Japan. The data on the number of pharmacists in each of 21 secondary medical areas, which are regional units providing in-hospital medical services, are obtained from the Ministry of Health, Labour, and Welfare [22]. Data on land area were obtained from Japan Medical Analysis Platform [23]. The data on population, aging rates (i.e. the rate of population aged ≧ 65), and estimated future population are obtained from the National Institute of Population and Social Security Research [8] for each medical area, as shown in Table 1. Hokkaido has a population of approximately 5.4 million; Sapporo area has the highest population of approximately 2.4 million, followed by Kamikawachubu (0.39 million population), and Minamioshima (0.38 million). In the less populated areas, the population is aging fast with the aging rates being more than 30% on average. For data on population distribution and geographical locations of the 21 secondary medical areas, see Fujiwara et al. [9]. The main outcome is “sufficiency rate” calculated by dividing the projected supply by the projected demand which are converted to the number of prescriptions. In this study, differences among prescriptions are not considered.

**Table 1:** Data on population and number of pharmacists in each medical area in Hokkaido [8, 22, 23]

Medical area	Population in 2015 [8]	Aging rate in 2015 (%) [8]	# of pharmacists in 2014 [22]	Estimated population in 2025 (change from 2015) [8]	Estimated population in 2040 (change from 2015) [8]	Land area (km^2^) [23]
Total	5,371,742	29.0	6234	5,016,554 (− 6.6%)	4,280,427 (− 20.3%)	78,454
Sapporo	2,375,449	25.2%	3039	2,377,341 (+ 0.1%)	2,218,734 (− 6.6%)	3540
Kamikawachubu	394,270	32.1	501	365,532 (− 7.3%)	306,101 (− 22.4%)	4238
Minamioshima	381,629	32.3	526	331,212 (− 13.2%)	252,822 (− 33.8%)	2671
Tokachi	343,436	28.8	343	325,611 (− 5.2%)	288,298 (− 16.1%)	10,828
Kushiro	236,516	30.4	258	208,707 (− 11.8%)	162,733 (− 31.2%)	5998
Hokumo	222,696	30.8	196	198,393 (− 10.9%)	157,087 (− 29.5%)	5542
Shiribeshi	215,522	35.6	255	178,117 (− 17.4%)	126,583 (− 41.3%)	4306
Higashiiburi	212,059	28.1	209	198,637 (− 6.3%)	169,906 (− 19.9%)	2340
Nishiiburi	189,696	29.4	204	164,447 (− 13.3%)	125,020 (− 34.1%)	1357
Minamisorachi	166,691	35.5	164	137,171 (− 17.7%)	96,651 (− 42.0%)	2562
Nakasorachi	108,970	37.9	114	89,175 (− 18.2%)	62,271 (− 42.9%)	2162
Nemutro	76,621	26.8	45	67,104 (− 12.4%)	52,154 (− 31.9%)	3533
Emmon	70,846	34.7	33	59,055 (− 16.6%)	42,381 (− 40.2%)	5148
Hidaka	69,015	31.5	73	56,314 (− 18.4%)	39,100 (− 43.3%)	4811
Soya	67,503	30.3	61	54,985 (− 18.5%)	38,020 (− 43.7%)	4626
Kamikawahokubu	66,591	34.4	57	55,731 (− 16.3%)	40,414 (− 39.3%)	4197
Rumoi	47,912	36.3	50	37,857 (− 21.0%)	24,856 (− 48.1%)	3446
Kitaoshimahiyama	37,279	35.6	30	29,650 (− 20.5%)	20,065 (− 46.2%)	2474
Kitasorachi	32,675	40.3	20	26,651 (− 18.4%)	18,318 (− 43.9%)	1067
Furano	36,550	40.4	42	32,597 (− 10.8%)	27,436 (− 15.8%)	2183
Minamihiyama	23,769	37.5	14	18,314 (− 23.0%)	11,477 (− 51.7%)	1423

### System dynamics modeling

SD is a simulation method based on stock-flow modeling, with “stock” explaining accumulation of resources (e.g. the number of pharmacists) and with “flow” explaining rates of change in “stock” variables (e.g. National exam pass rate). SD modeling is based on the actual causal relationships (e.g. career path of medical professionals), and the relationships are explained using the stock and flow of resources. A change in a “stock” in time *t* is explained by Eq. 1 [18].

1 $$\frac{d(\mathrm{stock})}{dt}=\mathrm{Inflow}\left(t\right)-\mathrm{Outflow}(t)$$

The merit of using SD is that dynamic relationships, such as feedback and non-linear relationships can be simulated. Recent trends in related parameters can also be reflected because parameter inputs in SD models are independent of past data, unlike in regression or time series analyses [17].

### Supply projection

**Fig. 1:**
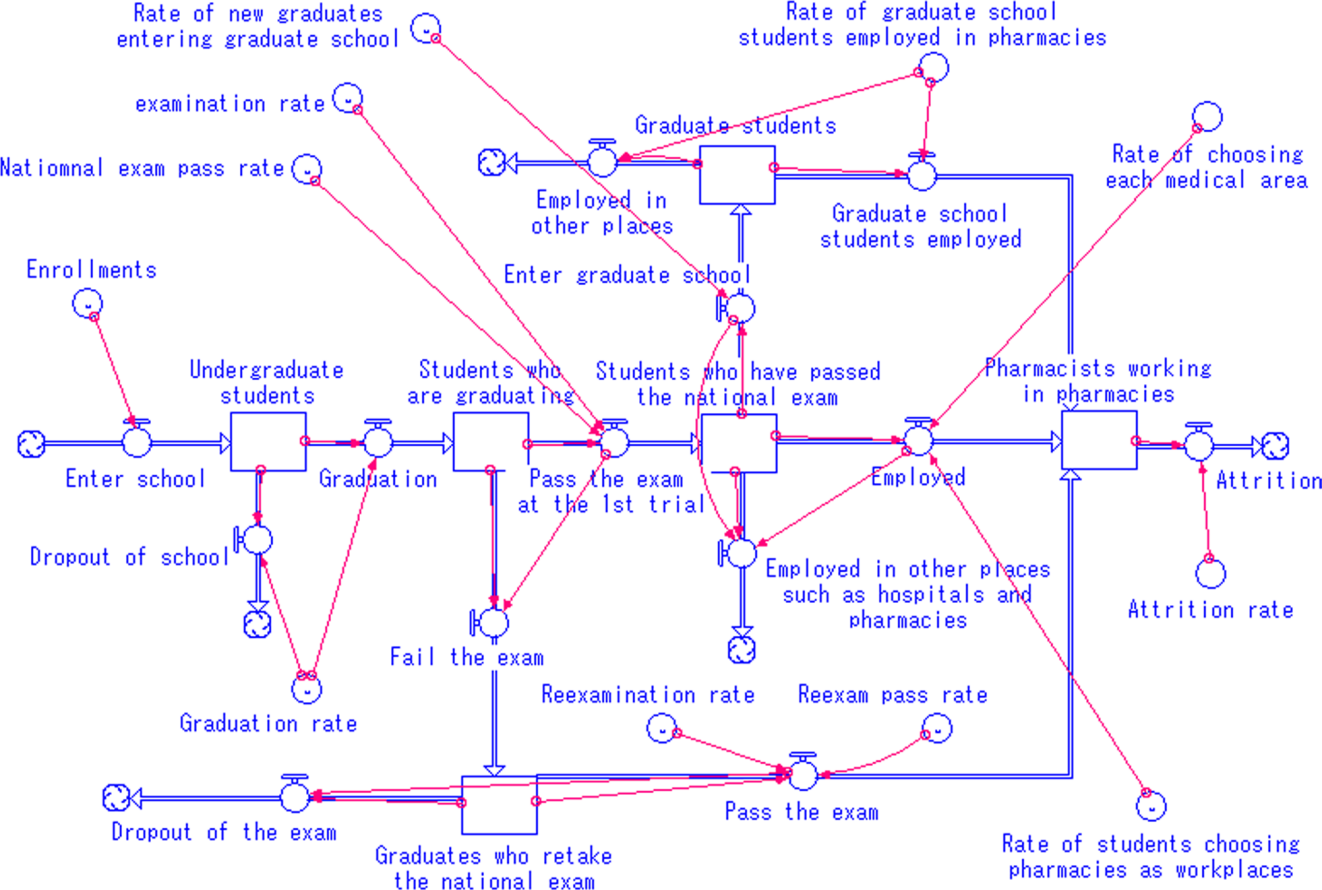
The supply projection model of pharmacists based on the career path. It shows the SD model to predict the number of pharmacists. The model structure was constructed based on the career path of pharmacists.

The projection model was created based on the pharmacists’ career path using the concept of system dynamics (Fig. 1). The squares in Fig. 1 refer to “stock” (e.g. the number of pharmacists), while the arrows refer to “flow” of “stock”. The circles decide the changing rate of the flow. The model structure was discussed with a pharmacist with more than 30 years of experience in pharmacies as well as researchers with experiences in SD modeling and simulation. Stella version 8.1.1 (isee systems, Lebanon, NH, USA) was used for the simulation. The list of parameters used in the model, input values, definitions (how each “stock” flow is calculated), and the sources of the parameters are shown in Table 2 [23–26]. The latest available data were used for the analyses. Since there are no data on the distribution of newly employed pharmacists, it is assumed that their distribution follows the distribution of currently employed pharmacists in each secondary medical area in the base case. The current distribution of pharmacists is shown in Table 1. In the model, students enroll in the 3 universities in Hokkaido and take the annual national exam for the pharmacist license 6 years after their enrollments. It is assumed that the students who fail the exam will take the exam the next year. After graduation, the students can study further in graduate schools or start working in hospitals, pharmacies, or companies as drug makers. It is also assumed that newly licensed pharmacists work who choose to work in pharmacies work in Hokkaido since Hokkaido is an insular prefecture, and moving to another prefecture is possible, but that accounts for little part. Since there are no data on the attrition rate of pharmacists in pharmacies, we have considered the pharmacists in both pharmacies and hospitals in estimating attrition rates. Attrition is defined as the gap between the number of newly employed pharmacists and the actual increase in the number of pharmacists (e.g., when 5 pharmacists are newly employed and the actual increase in the number of pharmacists is 3, it is assumed that 2 have left work). Even though attrition in the model includes any leaving from work such as quitting work or death as a whole, it can count the total flow of those leaving work.

**Table 2:** The list of parameters used in the supply projection model

Parameter	Definition	Input	Source	Year
Enrollments	–	399.8	24	2013–2017
Graduation rate	# of students taking the national exam in year *t*/# enrollments in year *t *− 6	0.962	24, 25	2015–2017
Examination rate	# of students taking the national exam in year *t*/# of students applying for the exam in year *t*	0.803	25	2015–2017
Reexamination rate	# of students re-taking the national exam in year *t*/# of students failing the exam in year *t *− 1	0.963	25	2015–2017
National exam pass rate	# of students passing the national exam in year *t*/# of students taking the exam in a year *t*	0.738	25	2015–2017
Reexam pass rate	# of students passing the national exam in year *t*/# of students re-taking the exam in year *t*	0.593	25	2015–2017
Rate of choosing pharmacies as workplaces	increase in # of pharmacists in pharmacies in year *t*/# of students passing the exam in year *t*	0.458	24	2013–2017
Rate of new graduates entering graduate schools after graduation	# of students entering graduate school in year *t*/# of students graduating in year *t*	0.025	24, 25	2013–2017
Rate of graduate school students employed in pharmacies	# of graduate school students employed in pharmacies after graduation /# of graduate school students	0.39	24, 25	2013–2017
Attrition rate (includes any leaving from work such as quitting work or death)	(# of students or graduates passing the national exam in year *t*) * (rate of choosing to work in pharmacies or hospitals)/(increase in # of pharmacists in pharmacies and hospitals)/(# of pharmacists in pharmacies and hospitals)	0.007	24–26	2012–2016
Rate of choosing each medical area	Distribution of new pharmacists in each area according to the current distribution of pharmacists (Table 1)	Table 1	23	2014

### Model validation

The model validity was tested according to previous studies [13, 19]. Since it is impossible to know how accurate the projected future number is, validity of SD model is tested using past data. In this study, we projected the number of pharmacists in 2012–2016, and compared the results with the actually published number of pharmacists. Rooted Mean Squared Errors (RMSE) of the number of pharmacists was used for the evaluation, as it is explained in Eq. 2, where let *n* be the number of samples, let *y* be the projected number of pharmacists, and let *y* with bar be the actually published number of pharmacists. RMSE of 0.1 is used as a threshold of evaluating validity of SD models [13].

2 $$\mathrm{RMSE}=\sqrt{\frac{{\sum }_{1}^{n}{\left[\frac{|{\stackrel{-}{y}}_{t-}{y}_{t}|}{{y}_{t}}\right]}^{2}}{n}}$$

### Demand projection

The demand for the pharmacists is projected based on the number of prescriptions. For this, future demographic changes are assumed to be the main drivers since Japan has an aging population; an increase in medical needs can be attributed to demographic changes [27]. Firstly, the data on the average number of prescriptions by age group are calculated from the population data [8] and the total number of prescriptions by age groups. The data for the total number of prescriptions by age include both the in-hospital pharmacy and other pharmacy prescriptions. Therefore, the number is adjusted by the rate of separation in dispensing and prescribing in Hokkaido [28]. The future demand is estimated by multiplying the average number of prescriptions by age group with the estimated future population by age group [8]. It was assumed that the number of prescriptions per person was constant during the analysis period.

Currently, approximately 80% of pharmacists work time was spent on prescriptions (i.e. making prescriptions, dispensing medication, and taking medication history) [20]. We defined “Demand coefficient” as the proportion of time spent on prescriptions in their total work time. The demand coefficient was set to use prescriptions as a proxy of pharmacists’ work. The demand coefficient was set as 0.8. Demand for pharmacists was calculated by dividing the total number of prescriptions by the Demand coefficient. That is, the demand for pharmacists is calculated by Eq. 3, where *i* refers to 5 year age group, and *p* refers to the number of prescriptions per person per year.

3 $${\text{Demand for pharmacists}} = \mathop \sum \limits_{i}^{n} ({\text{population}}_{i} \times p_{i} )/{\text{Demand coefficient}}$$

### Sufficiency evaluation

Firstly, the average per-day number of prescriptions made by one pharmacist is obtained by dividing the total number of prescriptions [28] by the number of pharmacists [22] and by the average number of working days in a year (253.8 days, obtained from a Ministry of Health, Labor, and Welfare survey [29]) (calculated as 17.64 prescriptions by one pharmacist in Hokkaido per day). Next, future supply in Hokkaido overall and each medical area is calculated by multiplying the average number of prescriptions made by one pharmacist per day with the number of full-time equivalent pharmacists. The projected supply in the number of prescriptions was compared to the projected demand. The future supply of pharmacists is evaluated using supply/demand ratio (sufficiency ratio). Sufficiency ratios larger than 1 mean supply surpasses demand. Considering possible differences in the number of prescriptions made by one pharmacist per day, sensitivity analyses are conducted by changing the number (17.6) to 15, and 25 (15-prescription scenario, and 25-scenario case, respectively). The case with 17.6 prescriptions per pharmacist per day is considered as the base case.

### Sensitivity analysis

Sensitivity analysis of the sufficiency ratio was conducted to estimate the effects of uncertainty or effects of expected changes in related parameters. For example, in the Japanese system there are both private schools and public schools for pharmacist licensure, and therefore the enrollments can be partly controlled. National exam rate can also be partly controlled through controlling the difficulty of the exam. On the other hand, parameters such as attrition rates and birth rates might change with uncertainty. Thus, it is desirable that the effects of these parameters on the demand/supply be estimated. Parameters for which sensitivity analyses were conducted, and the sensitivity ranges of those parameters are listed in Table 3.

**Table 3:** the parameters for which sensitivity analysis was conducted, and the sensitivity ranges

	Model inputs		
	Lower case	Base case	Upper case
The number of prescriptions per pharmacists per day	15	17.6	25
Birth rate and death rate for population projection	Future demand for pharmacists was calculated by using future population projection in low-birth and high death rate scenario, and high-birth and low-death rate scenario [8]		
National exam pass rate	0.638 (1st time) 0.493 (2nd time and after)	0.738 (1st time) 0.593 (2nd time and after)	0.838 (1st time) 0.693 (2nd time and after)
Enrollments	360	399.8	440
Attrition rate	0.002	0.007	0.012
The number of prescriptions per person by age group	0.9 × base case	Calculated by dividing the total number of prescriptions by age groups by the population in the same age groups	1.1 × base case

In this study, it was assumed that demand for pharmacists is driven by the population changes. We examined how sensitive the sufficiency ratio is to the population change by using population projection data in low-birth rate and high death-rate scenario and high birth-rate and low death-rate scenario estimated by National Institute of Population and Social Security Research [8].

The number of prescriptions per person by age group might change overtime, possibly through changes in patient behaviors and disease incidents. Therefore, the parameter was tested in the sensitivity analysis (Table 3).

Sensitivity analysis was also conducted for the national exam pass rate, enrollments, and attrition rates since the former two were partly politically controlled, where the latter one might change with uncertainty.

Diffusions of newly licensed pharmacists among the medical areas could change overtime. For example, the distribution of pharmacists has been concentrating in Sapporo, the capital of Hokkaido [5]. Therefore, scenario analyses were conducted for the diffusion. Three scenarios with different distributions were set (Table 4); (1) base scenario: newly licensed pharmacists were distributed in accordance with the current distribution of pharmacists, (2) recent trend scenario: newly licensed pharmacists were distributed reflecting the proportions of newly licensed pharmacists in 2012–2016, (3) previous trend scenario: newly licensed pharmacists were distributed reflecting the proportions of newly licensed pharmacists in 2006–2012.

**Table 4:** Scenario analysis: diffusions of newly licensed pharmacists

	Base scenario (reflected the current distribution in 2014) (%)	Previous trend scenario (reflected the trend in 2006–2012) (%)	Recent trend scenario (reflected the trend in 2012–2018) (%)
Sapporo	50.0	56.0	64.7
Kamikawachubu	8.2	10.7	8.6
Minamioshima	7.6	7.7	2.5
Tokachi	5.5	4.2	4.2
Kushiro	3.9	4.1	3.2
Hokumo	3.0	4.2	0.3
Shiribeshi	4.2	1.4	3.9
Higashiiburi	3.3	4.6	2.1
Nishiiburi	3.2	4.0	0.7
Minamisorachi	2.5	− 0.2	3.0
Nakasorachi	1.8	1.8	− 0.5
Nemuro	0.7	− 0.6	0.5
Emmon	0.6	0.8	3.3
Hidaka	1.2	0.5	1.9
Soya	0.8	0.2	− 0.8
Kamikawahokubu	1.0	0.6	1.5
Rumoi	0.7	− 0.2	0.1
Kitaoshimahiyama	0.4	0.7	− 0.4
Kitasorachi	0.3	− 0.2	− 0.1
Furano	0.7	− 0.8	1.2
Minamihiyama	0.2	0.4	0.1

## Results

### Model validation

**Fig. 2:**
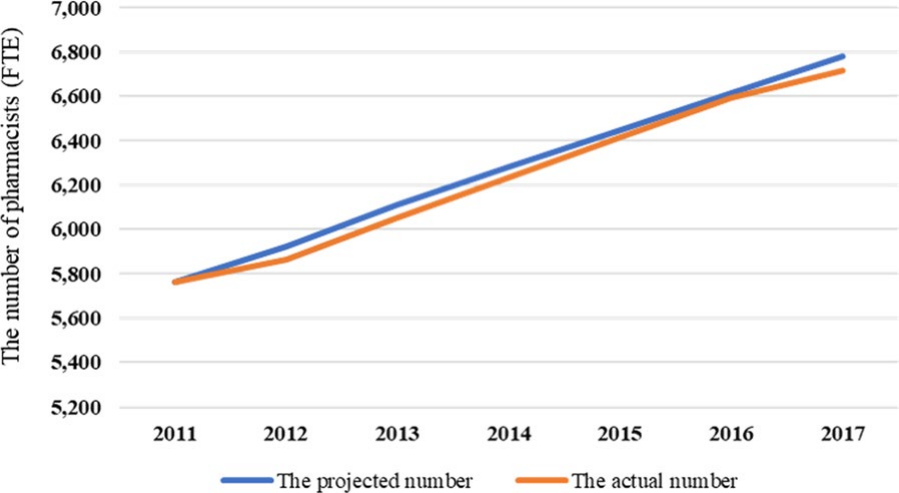
The projected number and the actual number of pharmacists in Hokkaido. The two numbers were compared for validation of the model

The projected number of pharmacists and the actual number of pharmacists were shown in Fig. 2. The calculated RMSE was 0.008.

### Supply projection

**Fig. 3:**
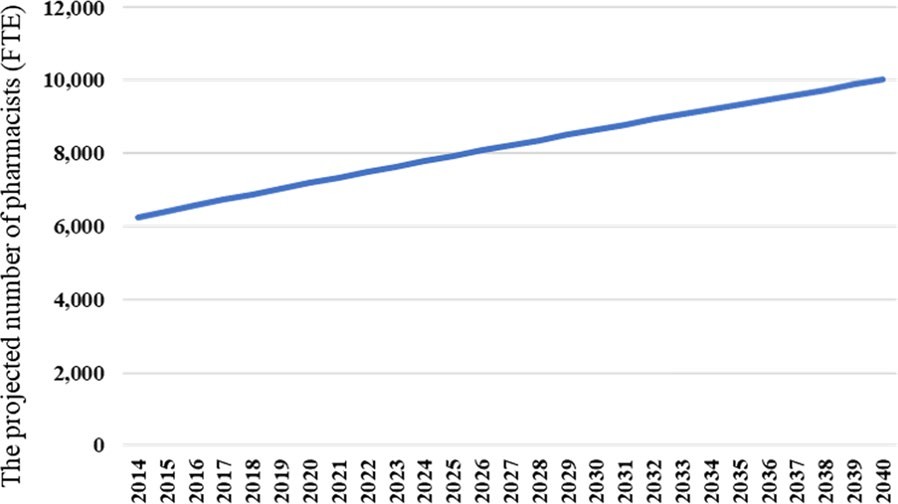
The projected number of pharmacists in pharmacies in Hokkaido. It shows the result of supply projection of pharmacists in Hokkaido from 2015 to 2040.

Figure 3 shows the results of the supply projection. The projected number of pharmacists is 7029 in 2025 and 10,008 in 2040, which are 1.24 times and 1.56 times that of 2015.

### Demand projection

**Fig. 4:**
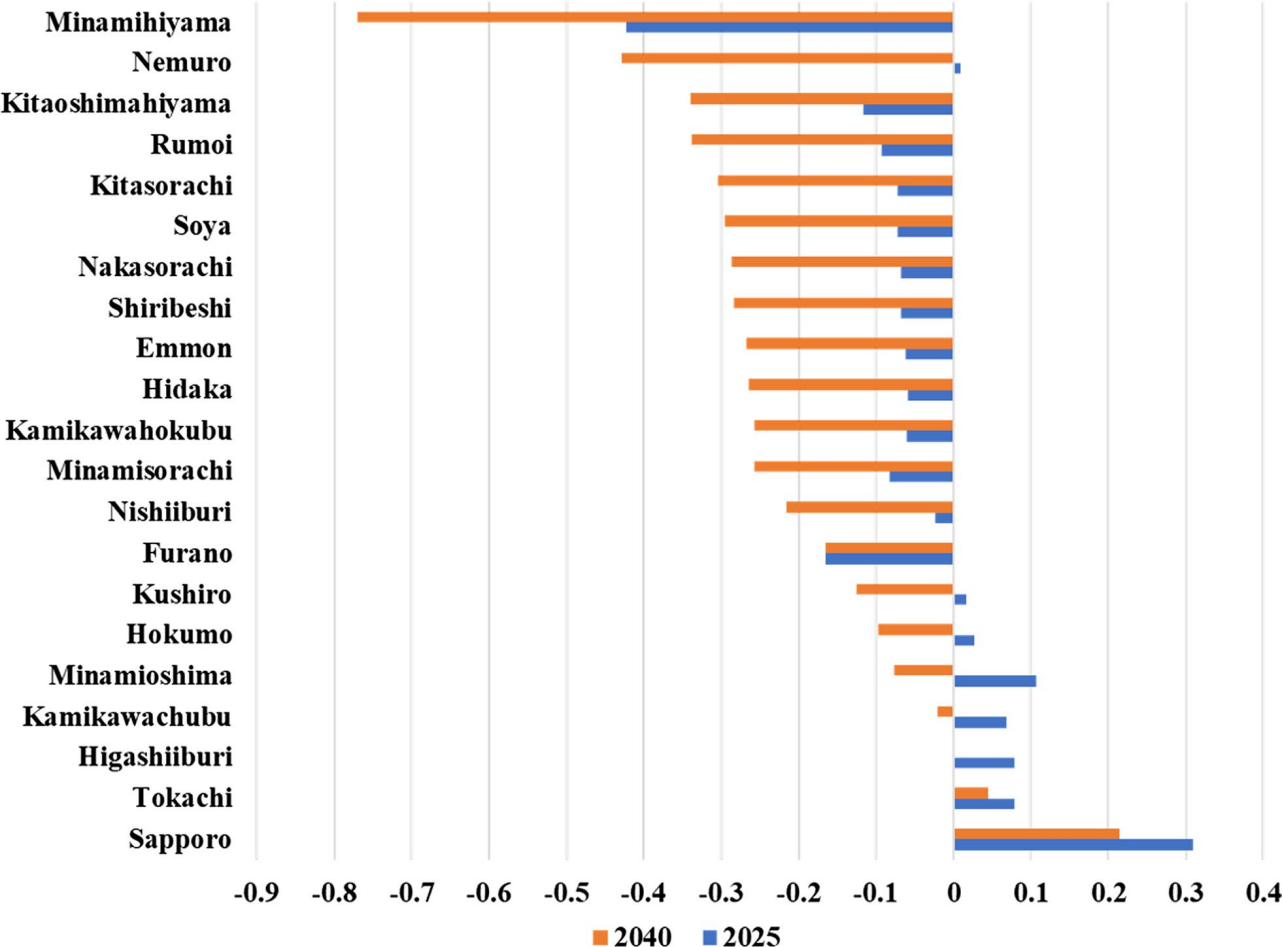
The estimated needs change from 2015 in each secondary medical area in Hokkaido. The result shows the trend that in populous medical areas such as Sapporo and Minamioshima, the demand will increase until 2025 and decrease afterwards. On the other hand, in rural areas such as Minamihiyama, Kitaoshimahiyama, Shiribeshi, the demand was expected to keep decreasing from 2015 to 2040.

The demand for pharmacists in Hokkaido overall is approximately 24 million in 2015, 27 million in 2025, and 24 million prescriptions in 2040. The demand trend for each secondary medical area and Fig. 4. During 2015–2025, the demand will increase in Minamioshima, Sapporo, Higashiiburi, Kamikawachubu, and Tokachi, most of which are populous areas. By 2040, the trend shows a decrease and the estimated demand in 2040 is less than that of 2015 for all medical areas except Sapporo. In Minamihiyama, Kitaoshimahiyama, Shiribeshi, Hidaka, Kamikawahokubu, Furano, and Rumoi, the demand is expected to decrease from 2015 to 2040.

**Fig. 5:**
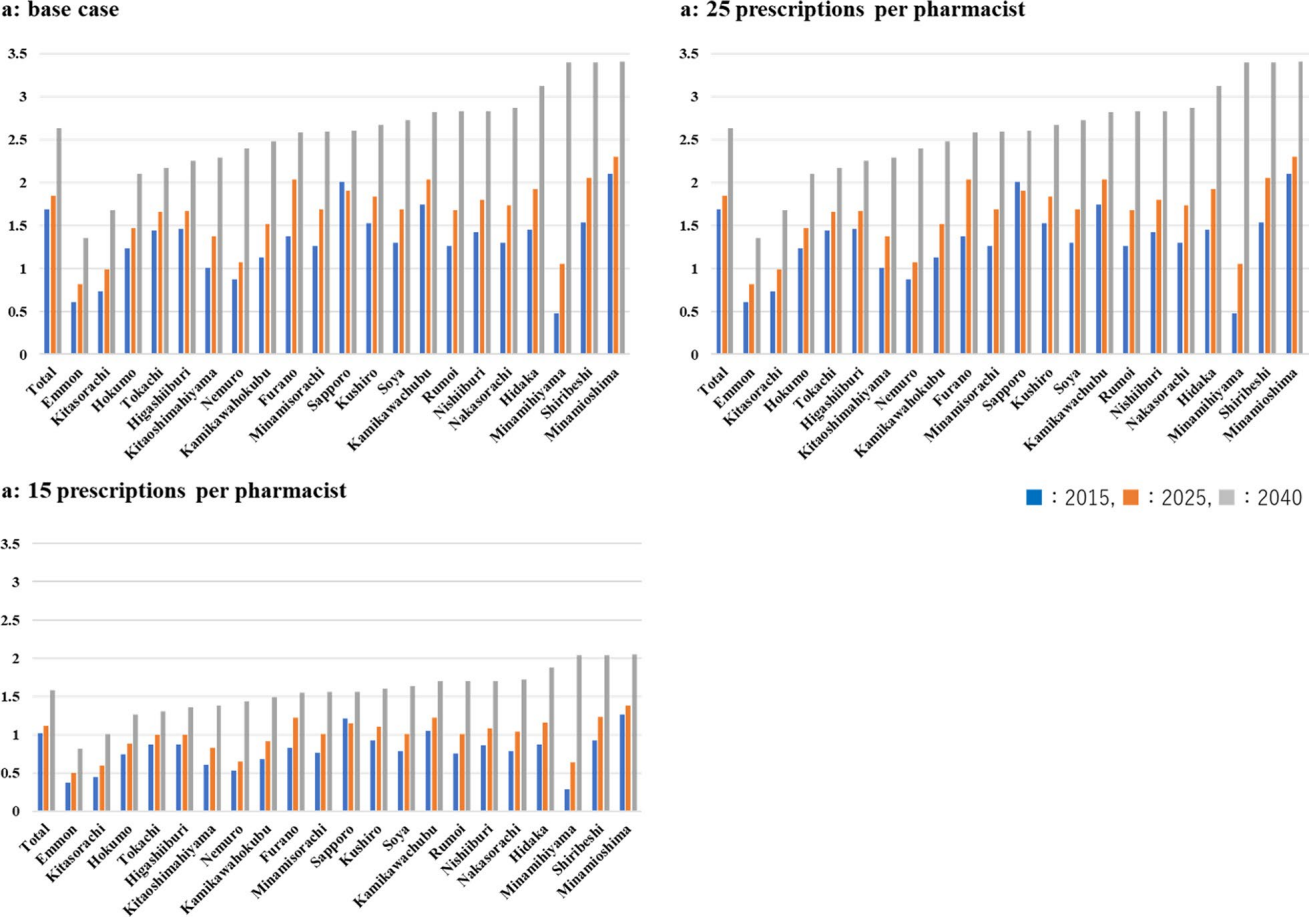
The results of the scenarios with different number of prescriptions per pharmacists per day. The estimated sufficiency ratios of pharmacists for each secondary medical area in Hokkaido in the scenarios with different number of prescriptions per pharmacists per day (**a**) 17.64, (**b**) 25, and (**c**) 15

### Sufficiency ratio

The sufficiency ratios for Hokkaido overall are 1.19 in 2015, 1.30 in 2025, and 1.85 in 2040, increasing rapidly after 2025 in the base case. The sufficiency ratios for each secondary medical area in the base case and in the 15-prescription scenario and 25-prescription scenario are shown in Fig. 5a–c, respectively. In 2015, the ratios are the highest in Minamioshima (1.48), Sapporo (1.41), and Kamikawachubu (1.23), while in less populated areas the ratios are below 1 [e.g. Minamihiyama (0.34), Minamisorachi (0.89), Nakasorachi (0.91), Kitasorachi (0.52), Kamikawahokubu (0.79), Rumoi (0.89), Soya (0.92), Hokumo (0.87), Emmon (0.43), and Nemuro (0.62)]. In Minahiyama, Kitasorachi, Emmon, and Nemuro, the ratios remain below 1 even when it is assumed that one pharmacist deals with 25 prescriptions per day. In these 4 medical areas, the sufficiency ratios remain below 1 in 2025 in the base case while, in Minamihiyama, the ratios surpass 1 in the 25-prescription case. In Sapporo, Minamioshima, Kamikawachubu areas, the ratios were more than 1 even when it is assumed that one pharmacist deals with 25 prescriptions per day.

**Fig. 6:**
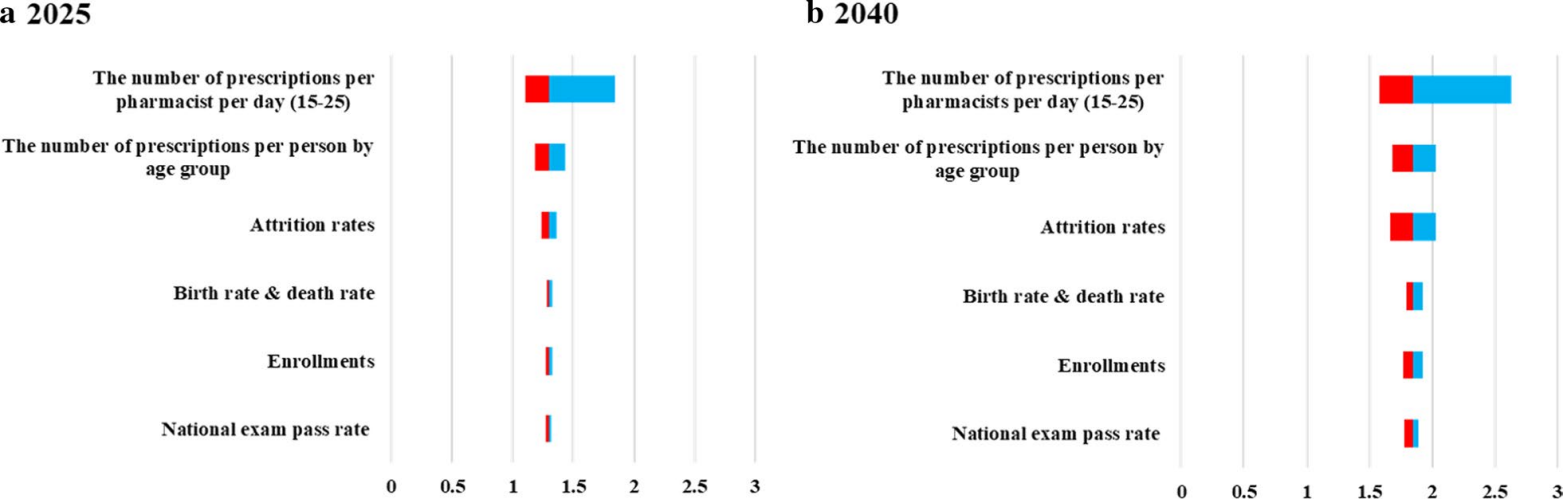
The results of sensitivity analyses of the sufficiency ratio in **a** 2025 and **b** 2040. In the sensitivity analyses, the values of the number of prescriptions per pharmacists per day, birth rate and death rate, attrition rates, enrollments, and national exam pass rate were changed according to the sensitivity ranges listed in Table 3

In 2040, the sufficiency ratios are more than 1 for all medical areas except Emmon (0.96). However, the sensitivity analysis result shows that in Emmon, the supply will be sufficient in the 25-prescription case. Conversely, the ratios are more than 2 in Minamioshima (2.41), Shiribeshi (2.40), Sapporo (2.36), Minamihiyama (2.40), Hidaka (2.20), Nishiiburi (2.00), and Nakasorachi (2.02) in the base case.

Figure 6 shows the results of sensitivity analyses. When the national exam pass rate was changed, the sufficiency ratio ranged from 1.27 to 1.32 in 2025, and 1.78 to 1.89 in 2040. When the enrollments were changed, the sufficiency ratio ranged from 1.27 to 1.33 in 2025, and from 1.77 to 1.93 in 2040. When the attrition rate was changed, the sufficiency ratio ranged from 1.24 to 1.36 in 2025, and from 1.67 to 2.03 in 2040. When birth rate and death rate were changed, the sufficiency ratio ranged from 1.28 to 1.33 in 2025, and from 1.80 to 1.92 in 2040. When the number of prescriptions per pharmacist per day was changed to 15 and 25, the sufficiency ratio ranged from 1.11 to 1.84 in 2025, and from 1.58 to 2.63 in 2040. When the number of prescriptions per person was changed, the sufficiency ratio ranged from 1.18 to 1.43 in 2025, and from 1.68 to 2.03 in 2040.

**Fig. 7:**
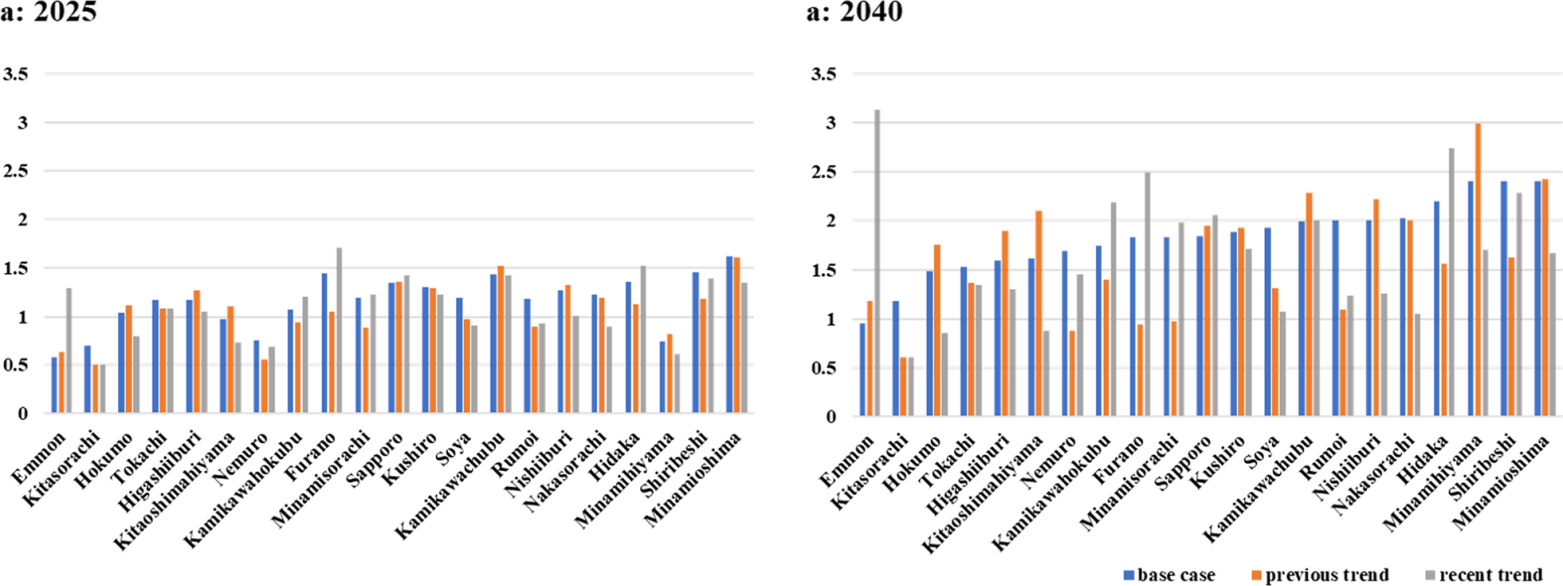
The results of scenario analyses for distributions of newly licensed pharmacists. It shows the results of scenario analyses for distributions of newly licensed pharmacists in **a** 2025 and **b** 2040. In the base case scenario, newly licensed pharmacists were distributed according to the current distribution. In the previous trend scenario, newly licensed pharmacists were distributed according to the increasing trend in 2006–2012. In the recent trend scenario, newly licensed pharmacists were distributed according to the increasing trend in 2012–2018.

Figure 7 shows the results of sufficiency ratios in the scenarios with different distributions of newly licensed pharmacists in the base scenario (Fig. 7a) and in the recent trend scenario (Fig. 7b). In the recent trend scenario, the sufficiency rates were higher than 1 in Emmon, Kamikawahokubu areas in 2025, where the sufficiency rates were lower than 1 in the base scenario. In 2040, the sufficiency ratios were more than 2 in some rural areas such as Shiribeshi, Emmon, Hidaka, Kamikawahokubu, and Furano in the recent trend scenario while the ratios were lower than 1 Hokumo Kitaoshimahiyama, and Kitasorachi in that scenario.

## Discussion

Supply of and demand for pharmacists in pharmacies in Hokkaido were estimated in this study using SD modeling to help determine their optimal distribution. The model was validated by the behavior test using the past data.

In 2025 and 2040, the number of pharmacists in Hokkaido is projected to be 1.24 times and 1.56 times, respectively, as that of 2015. Conversely, while demand is expected to be 1.13 times higher than that of 2015, it is expected to decrease after 2025, showing a different trend from the supply trend. The increase in the demand until 2025 can be attributed to demographic changes (i.e. aging of population). Conversely, the total population of Hokkaido is expected to decrease after 2025 and, hence, the increase in the number of elderly people is expected to be less rapid [8]. These demographic changes could decrease the demand for pharmacists after 2035.

The sufficiency ratio is approximately 1.19 in 2015 and 1.30 in 2025. Although it is difficult to determine the optimal sufficiency ratio, the current value (1.19) is not likely to indicate significant shortage or an oversupply when allowing for some range of uncertainty. Focuses should be on relative shortages, such as the distribution among specialties [15] and regions [17, 19].

Our results reveal that in 2015, although pharmacist supply tends to be sufficient for the populous medical areas, such as Sapporo, Minamioshima, and Kamikawachubu, there could be shortages in less populated areas, such as Minamihiyama, Kitasorachi, and Emmon. Morii et al. reported that in Hokkaido, pharmacists were as unequally distributed as physicians [5]. Their study used Gini coefficient as a measure of maldistribution, while this study used the sufficiency ratio, more specifically trying to figure out the extents of a surplus or a shortage is. The medical areas where the sufficiency ratios are below 1 are more aged (e.g. the aging rates are 37.5% in Minamihiyama, 40.3% in Kitasorachi, and 34.7% in Emmon areas) [8]. In these less populated areas, the demand peaks in 2015 and decreases thereafter. Our results indicated that there were shortages of pharmacists in less populated areas, such as Minamihiyama, Kitasorachi, and Emmon areas. However, since pharmacists are expected to promote the health of the local population, their distribution should meet the local population’s needs in time. In these medical areas, it is not likely that the pharmacist supply has room for expanding their work to home medical care and promoting self-medication. Compared to other prefectures, Hokkaido has larger medical areas, and therefore, its medical plan states that some medical services could be provided by more than one medical area working collaboratively [10]. It is possible that the shortages are covered by patients going to neighboring medical area (e.g. Minamihiyama to Minamioshima). It is also possible that the rate of separation in dispensing and prescribing and the number of prescriptions per pharmacist differ among the areas, which possibly cover the shortages. Although the universal rates were used in all the areas in Hokkaido due to lack of data, this simulation will be further improved with better availability of those data. An optimal supply should be considered for the medical areas where there are possible shortages of pharmacists [3].

Between 2015 and 2025, the demand was estimated to increase in Sapporo and Minamioshima. The increase was especially sharp in Sapporo area. The Ministry of Economy, Trade, and Industry reports that in regions where the aging rate is high, the demand for medical resources peaks early [25]. The same trends can be seen in the demand for pharmacists. In Sapporo and Minamioshima, the demand for pharmacists peaks later because the aging rates in those areas are not as high as that of the other areas. In contrast, the demand in Minamihiyama, Shiribeshi, Minamisorachi, Hidaka, Kamikawahokubu, Furano, and Rumoi areas would decrease from 2015 to 2040. This could be the result of the highly aged population in those areas. In 2025, since the sufficiency ratios remains below 1 for Kitasorachi and Emmon, the future supply in those areas should be reconsidered.

The results of the scenario analyses showed that in 2025, the sufficiency ratio in Emmon would surpass 1 in the recent trend scenario (Fig. 7). The result indicates if new pharmacists diffuse to the area at the recent pace, the demand will be met by 2025. Likewise, it is possible that reactions of pharmacist diffusion could occur in future to make up for the existing shortages. The results also indicated that the pharmacist sufficiency was more sensitive to its diffusion than the other parameters such as national exam pass rate and enrollments. It is important to estimate the optimal diffusion, and SD is a useful tool to estimate the effect of possible reactions on the pharmacist supply.

In 2040, the sufficiency ratios are nearly or more than 2 in 7 medical areas and more than 1 for all the medical areas except for Emmon (Fig. 5). After 2025, when the number of people over 75 is the maximum, the demand for pharmacists decreased rapidly (Fig. 4). In the 2025–2040 period, while the supply was projected to increase steadily, the demand was projected to decrease sharply, especially in less populated medical areas. This decrease is likely caused by a decrease in population. In those less populated areas, although the current supply is likely to be insufficient, there could be future oversupply if the number of pharmacists in those areas continues increasing at the current pace. Therefore, our results indicate that the optimal supply should be determined based on the estimated demand for each medical area at each point in time, that is, in order not to cause an oversupply. In less populated areas, a consideration should be made regarding the current sufficiency and the projected sufficiency.

This study considered the supply and demand for pharmacists working in pharmacies based on the number of prescriptions, the main aspect of pharmacists’ work. Demographic changes (i.e. aging of society and decreasing population) are the major drivers of an increase in medical needs [30] in Japan.

Sensitivity analyses were conducted to estimate how sensitive pharmacist demand/supply is to the related parameters. The sufficiency ratio was most sensitive to the number of prescriptions per pharmacists per day (Fig. 6), indicating changes in the roles of pharmacist will largely affect its sufficiency. It is likely that their roles will be expanded. Therefore, refinement of the model is necessary over time.

The effects of changes in enrollments, attrition rates, birth rate and death rate and the number of prescriptions per person by age were also estimated in the sensitivity analyses. These parameters could change with uncertainty or politically. Therefore, the results of great importance in estimating the effects of possible changes in these parameters.

SD modeling enables us to reflect on the recent trends in related parameters when projecting the quantum of resources in the future. Our methodology can be applied to other prefectures or regions in Japan. Application of supply/demand projection based on the number of prescriptions to other countries should be conducted carefully since pharmacists’ work and/or the medical system can differ among countries. The model structures should be considered with caution according to the settings. Although recent trends of parameters can be reflected in the simulation. At the same time, that holds a limitation and the simulation only reflects how the demand/supply will be with the current trends (e.g. we conducted the scenario analysis and tried to estimate the effects of changes in diffusion of new pharmacists. However, Nevertheless, it is difficult to completely tell what potential reaction of the system will be.) In addition, roles of pharmacists could also change over time (e.g. shift to home-healthcare). Therefore, repeated refinements will be necessary with changes in the related parameters continuously.

This study has several limitations. First, because of insufficient data, some parameters, such as the diffusion of newly employed pharmacists and attrition rates are assumed. For example, we assumed that the number of prescriptions per person is constant during the analysis period. Due to lack of data, it is assumed that the number of prescriptions among people over 75 does not differ among age groups. The demand was defined only by demographic changes, which has been thought to be a main driver of medical demand in Japan and does not consider factors such as chronic conditions and changing patterns of morbidity. Increases in the availability of data will increase the granularity of our analyses. However, efforts were made by conducting scenario analyses to estimate the effects of uncertainty. Second, the model structure was determined by one pharmacist who has more than 30-year clinical experience, and the researchers who have experiences in modeling studies for human health resources even though the behavior test confirmed the validity of the test. Third, there are several facts that were not considered in the model. For example, movements of pharmacists between the medical areas, and shifting of work from full-time to part-time, differences in the typical FTE in each medical area, and differences in the rate of separation in dispensing and prescribing, are not considered. Fourth, geographical accessibility was not considered in the analyses. Since pharmacists are expected to promote local people’s health, it is necessary that pharmacists work in people’s living areas. Therefore, future studies should focus on geographic accessibility to pharmacists in smaller regional units such as municipalities while this study focused on distribution of pharmacists in secondary medical units. Lastly, the demand projection was partly based on utilization of the services. It is possible that potential needs for the services do not lead to utilization because of lack of accessibility, or changes of patient behaviors. Therefore, evaluation in terms of needs will also be required in future studies.

## Conclusion

This study projected the future supply and demand for pharmacists in pharmacies and considered current and future sufficiency based on number of prescriptions to consider their optimal distribution. Sensitivity analyses of the sufficiency ratio were conducted to estimate the effects of changes parameters such as national exam pass rate, enrollments, attrition rates, the number of prescriptions per pharmacist, and diffusion of newly licensed pharmacists. The results revealed that the current supply is concentrated in populous medical areas, such as Sapporo, Minamioshima, and Kamikawachubu, while it is possible that less populated areas, such as Emmon, Kitasorachi, and Minamihiyama are short of pharmacists. The demand is projected to increase until 2025 and decrease thereafter. As a result, future supply would greatly surpass demand by 2040 in most medical areas. This result highlights the necessity of determining the optimal supply of pharmacists in order not to cause an oversupply after 2025, which is affected by demographic changes. The sensitivity analyses found that the sufficiency ratio was most sensitive to diffusion of newly licensed pharmacists and the number of prescriptions per pharmacist.

## Data Availability

The datasets used and/or analyzed during the current study are available from the corresponding author on reasonable request.
